# Improving Cardiac Insights: Harnessing Privacy-Preserving Record Linkage (PPRL) to Obtain, Link, and Enhance Data for a Healthier Australia

**DOI:** 10.23889/ijpds.v9i5.2514

**Published:** 2024-09-18

**Authors:** Philip Witowski, Windra Sulaiman, Mark Sipthorp, Adam Ismail, Beverley Phillips, Sharon Williams

**Affiliations:** 1 Centre for Victorian Data Linkage

## Objective

General Practices (GPs) collect vital information on health and disease in Australia, however much of this sensitive data is difficult to share for linkage purposes due to confidentiality and privacy concerns. PPRL techniques have presented an opportunity to work with GP’s, and better understand health conditions such as negative cardiac outcomes in Australia.

## Approach

We utilised two algorithms, GHRANITE and Bloom Filters, to hash 2.5 million records from over 100 GPs. Both approaches utilised the same basic principles: data custodians pass identifying data through an irreversible hashing algorithm, and generate a unique key for each record. The hashing is done at the practice level ensuring that original identifying patient information never leaves GP's premises. Hashed data can be sent elsewhere, and if the same algorithm is used on a different set of data, linkage can be performed on both sets to identify individuals without exposing identifying information.

## Results

OThe PPRL process involved encoding over 150 million records from diverse datasets, encompassing hospitals, housing, and other sources. Initial results indicated 70.90% linkage utilising GHRANITE, and 84.10% linkage utilising Bloom Filters. Moving forward, the goal is to provide the linked data back to GPs, enabling them to identify high-risk patients and implement targeted interventions or additional measures.

## Conclusion

Through the successful implementation of PPRL, the project has made strides in overcoming data sharing barriers while safeguarding confidentiality. By harnessing innovative algorithms, the initiative has paved the way for more insights into cardiac outcomes and the potential for proactive healthcare interventions.

